# Elevated Peripheral Visfatin Levels in Narcoleptic Patients

**DOI:** 10.1371/journal.pone.0002980

**Published:** 2008-08-20

**Authors:** Norbert Dahmen, Nina Manderscheid, Jana Helfrich, Petra B. Musholt, Thomas Forst, Andreas Pfützner, Alice Engel

**Affiliations:** 1 Department of Psychiatry, University of Mainz, Mainz, Germany; 2 ikfe GmbH, Mainz, Germany; University of Sydney, Australia

## Abstract

**Objective:**

Narcolepsy is a severe sleep disorder that is characterized by excessive daytime sleepiness, cataplexies and a tendency towards obesity. Recent discoveries indicate that the major pathophysiology is a loss of hypocretin (orexin) producing neurons due to immunologically mediated degeneration. Visfatin is a recently described proinflammatory adipokine. It is identical to the immune modulating pre-B-cell colony enhancing factor (PBEF). Our study examines the hypothesis that visfatin levels are altered in narcoleptic patients.

**Methods:**

For the analysis, a total of n = 54 patients (n = 18 males and n = 36 females) with the diagnosis of narcolepsy according to DSM-IV and the International Classification of Sleep Disorders were examined (BMI mean 30.3±5.5, age mean 52.5±16.1 years). As a control group 39 unrelated (n = 12 males and n = 27 females) healthy volunteers with no sleep disorder according to DSM-IV were included (BMI mean 28.5±4.6, age mean 51.1±13.6 years). Peripheral visfatin levels were measured using a commercial enzyme immunoassay kit with a measurement range from 0.1–1000 ng/ml. Narcolepsy symptoms, severity and frequency of symptoms as well as the total duration of various aspects of the symptomatology were assessed by unstructured and structured clinical interviews in including the Stanford Center for Narcolepsy Sleep Inventory.

**Results:**

Circulating visfatin was found to be significantly increased in HLA DR2 positive narcoleptic patients compared to controls.

**Conclusion:**

Taken together, our results add to the evidence of disturbed immunological regulation in patients with narcolepsy.

## Introduction

Narcolepsy is a sleep disorder that affects approximately 0.05% of the population in Western countries [1]. Typical symptoms include day time somnolence, cataplexies (emotionally triggered, sudden, involuntary loss of muscle tone), sleep related hallucinations, sleep paralysis, and automatic behavior [1]. In addition, narcoleptic patients are more obese than healthy controls [2], [3]. On a physiological level, the disorder has been conceptualized as a disturbance in the regulation of sleep, REM sleep and wake cycles [1]. Recently, abnormalities in the orexin neurotransmitter system have been shown to cause narcolepsy-like symptoms in orexin-knockout mice [4] as well as in narcoleptic canine breeds [5]. Furthermore, a deficiency in orexin levels was found in the liquor cerebro spinalis and to a much lesser extent in the peripheral blood of narcoleptic humans [6], [7], [8]. Orexinergic neurons originate from the lateral hypothalamus and reach out to most parts of the central nervous system including the brain stem. Orexin plays a crucial role in regulating and maintaining sleep/wakefulness states and energy homeostasis. In most cases, however, narcolepsy is not associated with abnormalities of orexinergic genes, and the cause(s) of narcolepsy are still not fully understood. However, narcolepsy is strongly associated with the major histocompatibility class II haplotype HLA DRB1*1501-QB1*0602 [9], [10]. Clinical trials using intravenous immunoglobulin infusions in recent onset narcolepsy with cataplexy have led to improvement in cataplexy in some patients [11]. Despite the fact that specific antibody markers for narcolepsy are still elusive, an autoimmune-mediated mechanism is considered the most probable etiology for narcolepsy and the elucidation of immune markers is one of the top priorities in narcolepsy research [12], [13]. A number of studies have focused on cytokines. Vgontzas et al., 1997, reported elevated TNF-α plasma in narcoleptics based on the analysis of 11 narcoleptics and 10 controls [14]. Hinze-Selch, in an attempt of replication in nine narcoleptics and controls reported in 1998 that mitogen-stimulated monocytes of narcoleptics secreted more IL-6 than control monocytes, whereas no TNF abnormalities were found [15]. Okun et al., 2004, measured plasma levels of TNF-α and IL-6 in 39 narcoleptic patients and in 40 controls and confirmed significantly higher TNF-α and IL-6 levels in the narcoleptics [16]. Himmerich et al., 2006 measured TNF-α and its soluble receptor in 30 narcoleptics and 120 controls and found elevated levels of the TNF-receptor but not of TNF itself [17]. Indirect support for an involvement of cytokines in the pathophysiology of narcolepsy comes from a number of molecular genetic studies, including one of our own group in which variants of TNF were associated with narcolepsy [18], [19], [20].

Fukuhara et al. (2005) identified a molecule that is expressed at much higher levels in visceral fat than in subcutaneous fat [21]. This molecule, denoted visfatin to indicate its abundance in visceral fat, turned out to have been already identified as a growth factor for early B cells called pre-B cell colony-enhancing factor (PBEF), a 52-kilodalton cytokine also expressed in lymphocytes. Visfatin exerts insulin-mimetic effects in cultured cells and lowers plasma glucose levels in mice [21]. Visfatin dose-dependently upregulates the production of the pro- and anti-inflammatory cytokines IL-1, IL-1Ra, IL-6, IL-10, and TNF in human monocytes [22]. These cytokines play a substantial role in a wide range of infectious and inflammatory diseases including narcolepsy [16], [17], [23]. Visfatin has been linked to several inflammatory disease states such as acute lung injury and rheumatoide arthritis [24]. In addition, Visfatin is considered to be a putative biomarker for metabolic syndrome. However, therapeutic intervention with drugs known to alter insulin resistance and dyslipidemia did not result in any change in circulating visfatin levels in non-diabetic patients with elevated risk for macrovascular disease [25]. The role of visfatin in the pathophysiology of the metabolic syndrome is still under investigation.

To test the hypothesis that visfatin levels are altered in narcoleptics we compared peripheral visfatin levels of 54 narcoleptic patients with those of 39 controls.

## Methods

### Patients

This study is part of a broader effort to clinically and genetically characterize narcolepsy patients. For the present analysis, a total of n = 54 patients (n = 18 males and n = 36 females) with the diagnosis of narcolepsy according to DSM-IV and the International Classification of Sleep Disorders [26] were examined. Patients were all unrelated and were either from the Department of Psychiatry, Mainz, or recruited with the help of the Deutsche Narkolepsie-Gesellschaft, a nationwide German patient organization. Most narcoleptic patients visited our department to participate in the research program. Others who were unable or unwilling to come but eager to participate were visited by a member of the research team at home. Total duration of narcolepsy symptoms, severity of daytime sleepiness and frequency (0 = more than a year ago, 1 = within the past year, 2 = within the past month, 3 = within the past week, 4 = within the past 24 hours) of the symptoms cataplexies, automatic behaviour, hallucinations and sleep paralysis were assessed by unstructured and structured clinical interviews in including the Stanford Center for Narcolepsy Sleep Inventory (http://med.stanford.edu/school/Psychiatry/narcolepsy/sleepinventory.pdf). Patients were only enrolled into the study when unambiguous cataplexies in addition to severe daytime sleepiness were reported and when sleep laboratory confirmation of the diagnosis was present in the medical records. To exclude symptomatic narcolepsies, the medical history was assessed and a neurologic examination was performed. As a control group 39 unrelated (n = 12 males and n = 27 females) healthy volunteers with no sleep disorder according to DSM-IV were included. In order to maximise similarities in lifestyle and habits between patients and controls controls were either spouses or genetically unrelated aquaintances of the patients. Absence of narcolepsy or other major sleep disorders was checked by clinical interview and by the administration of the Stanford Center for Narcolepsy Sleep Inventory. Blood samples were collected in patients and controls after a 12-hour fasting period in the morning between 8:00 and 10:00 am. Samples were immediately centrifuged, cooled with dry ice and stored at −80°C until the visfatin measurements. An additional anticoagulated blood smple was taken for DNA preparation (HLA-testing).

All participants were of Caucasian origin. All participants gave written, informed consent. No participation fees were paid. The study design was approved by the local Ethics Committee of the Federal Chamber of Physicians of Rhineland-Palatinate. For a general characterisation of patients and controls, please see table 1.

**Table 1:** Characterisation of patients and controls.

Characteristics	Narcoleptics	Controls	p-values
*n*	*54*	*39*	
Males	18 (33.3%)	12 (30.8%)	n.s.
Females	36 (66.6%)	27 (69.2%)	n.s.
**Age (years)**	52.5±16.1	51.1±13.6	n.s.
(males)	60.4±14.7	50.3±13.7	n.s.
(females)	48.5±15.5	51.5±13.7	n.s.
**BMI (kg/m^2^)**	30.3±5.5	28.5±4.6	n.s.
(males)	31.1±4.9	29.5±4.0	n.s.
(females)	29.8±5.8	28.0±4.9	n.s.
Duration of narcolepsy (years)	32.8±17.9	n.a	
Age of onset (years)	20.1±11.9	n.a.	
**Symptoms**			
Daytime somnolence	54 (100%)	n.a.	
Cataplexies	54 (100%)	n.a.	
Hallucinations	45 (83%)	n.a.	
Sleep paralysis	34 (63%)	n.a.	
Automatic behavior	41 (76%)	n.a.	
**HLA DR2 pos.**	46 (85%)	9 (23%)	
(males)	16 (89%)	3 (25%)	
(females)	30 (83%)	6 (22%)	

n.s. = not significant; n.a. = not applicable or not measured

### Measurements

Visfatin levels were measured using an enzyme immunoassay kit from Phoenix Pharmaceuticals (Phoenix Europe GmbH, Karlsruhe, Germany) with a measurement range from 0.1–1000 ng/ml. This enzyme immunoassay kit is designed to detect a specific peptide and its related peptides based on the principle of “competitive” enzyme immunoassay.

The detection range was 0.1–1000 ng/ml with an inter-assay variation of less than 14% and intra-assay variation less than 5% according to the manufacturer.

The unknown concentration in samples can be determined by extrapolation to the standard curve.

All Patients and controls were HLA typed by standard PCR-SSO using a commercial kit (Dynal RELI SSO HLA-DRB1 Test; Invitrogen GmbH, Karlsruhe, Germany).

### Statistical Analysis

Descriptive results of continous variables are expressed as means±SD for Gaussian variables and as median and interquartile range for non-Gaussian variables. Group differences were tested with the Mann-Whitney-U-Test and the Kruskal-Wallis-Test. For the assessment of correlations, Spearman correlation coefficients were calculated. All analyses were two-tailed and conducted with SPSS software (version 12.0 for Windows). P-values of p<0.05 were considered to be statistically significant.

## Results

Mean visfatin concentrations were elevated in narcoleptic patients in comparison to age and BMI matched controls. The median was 33.7 ng/ml in narcoleptics (interquartile range 19.7–54.4) vs. 19.4 ng/ml in the controls (interquartile range 15.3–31.5; p = 0.036).

Visfatin values did not differ between women and men. The median for all women (narcoleptic patients and controls) was 26.1 ng/ml (interquartile range 15.3–50.4) and for all men was 25.5 ng/ml (interquartile range 17.3–58.3; p = 0.642). The visfatin values for the female narcoleptics were 38.3 ng/ml (interquartile range 20.7–54.8) and 27.9 ng/ml for the narcoleptic males (interquartile range 17.3–58.4; p = 0.582) and for the control women 19.4 ng/ml (women, interquartile range 14.7–28.4) and 21.2 ng/ml for the male controls (interquartile range 17.4–68.6; p = 0.327).

**Figure 1:**
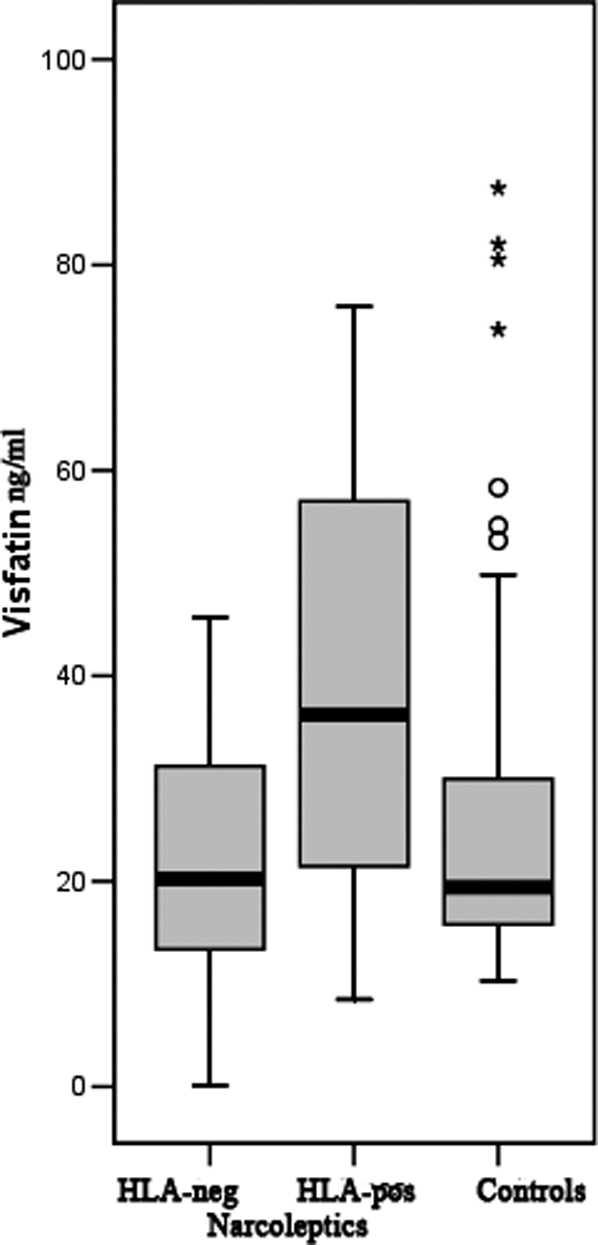
Boxplot comparing controls with narcoleptics, outliers above 100 ng/ml (one individual of the control group, five narcoleptic patients) are not depicted.

Closer examination of the data revealed that the group difference between narcoleptics and controls was mainly driven by the HLA DR2 positive narcoleptics (BMI 31.1±5.1; p = 0.51 compared to controls; age 54.2±16.2; p = 0.23 compared to controls). The median of the HLA DR2 positive narcoleptics was 36.2 ng/ml (interquartile range 21.0–57.5) vs. 20.2 ng/ml in HLA-neg. narcoleptics (interquartile range 12.6–35.2; p = 0.036) vs. 19.4 ng/ml in the controls (interquartile range 15.3–31.5; p = 0.010; figure 1). No differences between HLA positive and negative controls were apparent. The median of the HLA DR2 positive controls was 19.4 ng/ml (interquartile range 12.7–40.8) vs. 20.4 ng/ml in HLA-neg. controls (interquartile range 16.0–36.1; p = 0.756).

After the stratification in HLA DR2 positive and negative patients, there was again no difference between men and women: The median for HLA-pos. women was 44.2 ng/ml (interquartile range 25.1–57.5). The median for HLA-pos. men was 27.9 ng/ml (interquartile range 17.3–95.9; p = 0.321). The median for HLA-neg. woman was 17.7 ng/ml (interquartile range 8.9–28.9). The median for HLA-neg. men was 29.5 ng/ml (interquartile range 20.0–39.1; p = 0.643).

Visfatin was not correlated with age or BMI, neither in the HLA DR2 positive narcoleptic patients (age: corr. coeff. 0.062, p = 0.684; BMI: corr. coeff. 0.044, p = 0.772), in the HLA DR2 negative naroleptic patients (age: corr. coeff. 0.238, p = 0.570; BMI: corr. coeff. 0.204, p = 0.629) nor in the control group (age: corr. coeff. 0.316, p = 0.050; BMI: corr. coeff. −0.155, p = 0.346).

Finally, we calculated correlations between visfatin levels and different measures of the frequency and severity of narcoleptic symptoms, with the age of onset, the BMI and the duration of narcolepsy. Unexpectedly, we found a sifgnificant correlation between the self-reported frequency of cataplexies and visfatin levels in the HLA-DR2 positive male narcoleptics, but not in the HLA DR2 positive female narcoleptics. All other correlations, including those calculated with the HLA DR2 negative patients (data not shown) were non-significant (see table 2).

**Table 2:** Correlations between visfatin levels of HLA-pos. narcoleptic patients and clinical findings.

Clinical findings	all patients	male (n = 16)	female (n = 30)
	Corr.Coeff (Sig.)	Corr.Coeff (Sig.)	Corr.Coeff (Sig.)
Age of onset	−0.130 (0.388)	−0.348 (0.187)	−0.106 (0.578)
BMI	0.044 (0.772)	0.450 (0.080)	−0.125 (0.511)
Duration of disease	0.054 (0.724)	0.231 (0.389)	0.051 (0.791)
Score of daytime sleepiness (ESS)	−0.046 (0.769)	0.169 (0.580)	0.005 (0.978)
Frequency of cataplexy	0.322 (**0.033**)	0.780 (**0.001**)	0.157 (0.406)
Frequency of hallucination	−0.065 (0.675)	−0.110 (0.709)	0.047 (0.807)
Frequency of sleep paralysis	0.108 (0.487)	0.402 (0.154)	0.021 (0.914)
Frequency of automatic behaviour	−0.135 (0.412)	−0.075 (0.828)	−0.103 (0.602)

## Discussion

In our analyses, visfatin levels were significantly higher in HLA DR2 positive narcoleptic patients than in BMI- and age-matched controls. This result corroborates previous findings, that different measures of immunological activity are elevated in narcoleptics. A link between narcolepsy and altered immune functions has been hypothesized ever since the tight association between narcolepsy and the HLA-system had been recognized (for an excellent review see Mignot et al., 1995 [27] and Black 2005 [12]). The TNF-α system is of additional specific interest because it directly interacts with the visfatin system. By incubating fat tissue with TNF, Hector et al., 2007, demonstrated that TNF is a strong inductor of visfatin mRNA secretion and thus it appears conceivable that elevated TNF levels in narcoleptic patients contribute to the elevated visfatin in the present study [28]. On the other hand, visfatin is able to stimulate secretion of different cytokines such as interleukin beta, TNF and Il-6 and thus is considered immunomodulating and proinflammatory (Moschen et al., 2007) [22].

Human narcolepsy may have multiple etiologies and may develop at least partially on a genetic background. The high incidence of human leukocyte antigen (HLA)-DR2 haplotypes among narcoleptics suggests that an autoimmune process is involved in the pathophysiology of the disease. A relationship between narcolepsy and a deficiency of the peptide neurotransmitter orexin (hypocretin) has been established [29]. One hypothesis is that narcolepsy is an autoimmunologic disease that leads to the specific degeneration of hypothalamic hypocretin-containing cells. The observation of increased visfatin levels in narcoleptics is in good accordance with this thesis, because elevated levels of visfatin were also found in other autoimmune diseases such as as rheumatoid arthritis [30] and diabetes type I [31]. Moreover, elevated visfatin has been found in conditions that involve acute immunological activation such as colorectal cancer and acute lung injury (ALI) [32], [33].

In our study, the elevation of visfatin was only found in HLA DR2 positive narcoleptics, who constitute the vast majority of all cases, but not in HLA DR2 negative patients, whose visfatin levels were not distinguishable from the visfatin levels of the controls. This observation fits excellently in the theroretical framework described above because it is mainly the HLA DR2 positive patients who show diminished orexin levels whereas HLA DR2 negative patients tend to display orexin levels comparable to those of controls [34]. Accordingly, the autoimmune hypothesis of narcolepsy might be true only for the HLA DR2 positive or the central orexin deficient form. Therefore, the visfatin results support existing evidence [34], including molecular genetic evidence from our own group [27] that HLA DR2 positive and negative narcolepsy may represent different pathophysiological entitites leading to a common clinical appearance. In our study, as in previous studies [20], [35], [36], [37], [38], [39], [40], there was no correlation between age, BMI or gender and visfatin levels. The finding of a correlation between visfatin levels and frequencies of cataplexies in male but not female HLA DR2 positive patients was rather unexpected. Currently, we can not offer an evidentiary explanation. However, the issue of sexual dimorphisms in narcolepsy has previously been raised in the context of potentially cataplexy relevant neurotransmitter metabolisation [41].

In summary, our study adds to the growing evidence of subtle signs of immunological dysfunction in narcoleptic patients. Because our study is the first study measuring visfatin in narcoleptic patients, independent replication is necessary before considering the findings conclusive.
